# MCRBM–CNN: A Hybrid Deep Learning Framework for Robust SSVEP Classification

**DOI:** 10.3390/s25247456

**Published:** 2025-12-08

**Authors:** Depeng Gao, Yuhang Zhao, Jieru Zhou, Haifei Zhang, Hongqi Li

**Affiliations:** 1 School of Yonyou Digital Intelligence, Nantong Institute of Technology, Nantong 226001, China; gaodepeng@ntit.edu.cn (D.G.); 22263100@ntit.edu.cn (J.Z.);; 2 School of Software, Northwestern Polytechnical University, Xi’an 710000, China

**Keywords:** SSVEP classification, multi-channel restricted Boltzmann machine, convolutional neural network

## Abstract

The steady-state visual evoked potential (SSVEP), a non-invasive EEG modality, is a prominent approach for brain–computer interfaces (BCIs) due to its high signal-to-noise ratio and minimal user training. However, its practical utility is often hampered by susceptibility to noise, artifacts, and concurrent brain activities, complicating signal decoding. To address this, we propose a novel hybrid deep learning model that integrates a multi-channel restricted Boltzmann machine (RBM) with a convolutional neural network (CNN). The framework comprises two main modules: a feature extraction module and a classification module. The former employs a multi-channel RBM to unsupervisedly learn latent feature representations from multi-channel EEG data, effectively capturing inter-channel correlations to enhance feature discriminability. The latter leverages convolutional operations to further extract spatiotemporal features, constructing a deep discriminative model for the automatic recognition of SSVEP signals. Comprehensive evaluations on multiple public datasets demonstrate that our proposed method achieves competitive performance compared to various benchmarks, particularly exhibiting superior effectiveness and robustness in short-time window scenarios.

## 1. Introduction

Brain–computer interface (BCI) technology has emerged as a transformative tool for establishing direct communication between the brain and external devices, bypassing conventional neuromuscular pathways [1]. Based on signal acquisition methods, BCIs can be categorized into invasive, partially invasive, and non-invasive types. While invasive and partially invasive approaches can obtain high quality signals through implanted electrodes, they are associated with risks of infection and high costs. Non-invasive BCIs, which record electroencephalogram (EEG) signals via scalp electrodes, have become the mainstream research direction due to their operational convenience, high safety, and low cost [2].

Among various EEG paradigms, the steady-state visual evoked potential (SSVEP) represents one of the most promising BCI modalities, characterized by a high signal-to-noise ratio, considerable classification accuracy, and minimal user training requirements [3]. The SSVEP signal originates from the brain’s rhythmic response triggered by the human eye’s fixation on specific frequency flickering stimuli, and is often used to construct multi-instruction BCI systems. By guiding users to fixate on targets with different frequency light sources, the mapping of signal instructions can be achieved, thereby supporting multi-option interaction operations. However, the SSVEP signal is susceptible to noise, artifacts, and other brain activities, which poses challenges for signal decoding and classification. Therefore, developing robust and highly generalized signal analysis and classification methods are key factors for improving the performance of BCI systems.

Recently, deep learning has achieved remarkable results in different fields, such as computer vision and speech recognition, and has been gradually introduced into EEG signal processing tasks [4]. Compared with traditional methods, deep learning can automatically learn the latent structure of data and directly extract discriminative high-level features from the original signals, thereby reducing manual intervention and dependence on domain knowledge, and improving classification accuracy. In SSVEP classification, a convolutional neural network (CNN) demonstrates outstanding capabilities for feature extraction and pattern recognition, which can extract local and global temporal–spatial features through multi-layer convolution, and has become a research hotspot [5]. In parallel, generative models, like the restricted Boltzmann machine (RBM), offer a powerful framework for unsupervised feature learning. Their multi-channel extension, the multi-channel RBM (MCRBM), is especially promising for EEG, as it can explicitly model inter-channel dependencies to uncover richer latent representations [6]. Li et al. [7] designed a spatial–temporal discriminative RBM for single-trial event-related potential (ERP) detection, which jointly captures robust spatial and temporal features and achieves a state-of-the-art performance. Despite these individual strengths, a synergistic integration of MCRBM and CNN for SSVEP classification remains largely unexplored. The current literature lacks comprehensive studies that effectively combine the robust, unsupervised feature learning of MCRBM with the superior discriminative power of CNNs, leaving a gap in both model architecture design and parameter optimization guidance.

To bridge this gap, we propose a novel hybrid deep learning framework, MCRBM–CNN, which seamlessly integrates a multi-channel restricted Boltzmann machine-based feature extractor with a CNN-based classifier. This architecture is designed to first learn informative, channel-wise representations in an unsupervised manner, and then further abstract these features into highly discriminative spatiotemporal patterns for accurate classification. Extensive evaluations on public benchmarks demonstrate that our model achieves competitive performance, exhibiting pronounced advantages in challenging short-time window scenarios, thus offering a robust and efficient solution for practical SSVEP-based BCIs.

## 2. Related Works

### 2.1. Traditional Feature Extraction and Classification

Traditional methodologies for SSVEP classification predominantly rely on signal processing and statistical analysis techniques, which can be categorized into several core types [8].

(1) Methods based on a frequency domain analysis. This type of method takes the Fourier transform as the core and extracts the main frequency and harmonic components of SSVEP through frequency analysis. A power spectral density analysis (PSDA) uses the fast Fourier transform to map EEG signals from the time domain to the frequency domain, then calculates the energy distribution at each frequency point to identify the stimulus targets that the subject was focusing on [9]. This method is theoretically mature and easy to implement, but it is rather sensitive to the time window length of the signal and the electrode configuration, and is difficult to handle nonlinear and non-stationary features. In addition, the discrete Fourier transform (DFT) can also be used for SSVEP classification [10]. By estimating the phase information at different frequencies, DFT can improve the accuracy of spectral matching. Due to the high requirements for time windows in frequency domain analysis methods, it is difficult to adapt to a short-time window or real-time processing demands.

(2) Methods based on signal decomposition. This type of method captures the multi-scale time-frequency characteristics of the original EEG signals by decomposing them into multiple components. Wavelet transform (WT) is a commonly used multi-resolution analysis tool that can analyze the local characteristics of signals in terms of time and frequency. Rejer et al. [11] used WT to detect the main frequency of SSVEP. Heidari and Einalou [12] utilized the discrete wavelet transform to extract local features by convolving signals in different frequency bands. The empirical mode decomposition (EMD) method reconstructs the components of the signal at different frequencies by iteratively extracting a set of intrinsic mode functions [13]. Ensemble empirical mode decomposition introduces white noise to enhance stability [14]. Multidimensional empirical mode decomposition synchronously decomposes multi-channel signals to enhance the time-frequency alignment capability among different channels [15]. Moreover, some researchers have also attempted to combine EMD with canonical correlation analysis to enhance the ability of feature discrimination [16]. The signal decomposition method has high computational complexity, poor real-time performance, and is greatly affected by parameter selection.

(3) Methods based on spatial filtering. The spatial filtering can enhance the target frequency components by integrating multi-channel EEG signals, improving the classification performance [17]. For example, minimum energy combination constructs the projection signal with the minimum energy at the target frequency to achieve target detection [18]. The common spatial pattern maximizes the variance differences between different classes by constructing spatial filters, thereby extracting discriminative features [19]. Although such methods work well in some scenarios, their performance may decline when the signal window is short or they are disturbed by noise. The spatial filtering method may lose some useful information during the transformation process, which affects the final classification effect.

(4) Methods based on canonical correlation analysis. Canonical correlation analysis (CCA) is a statistical correlation analysis method. Its basic idea is to construct a set of reference sine or cosine signals, conduct typical correlation analysis with EEG signals, and select the frequency corresponding to the maximum correlation as the classification result [20]. Since it does not rely on training and has high computational efficiency, it has become one of the most common methods in SSVEP classification. In recent years, several improved versions of CCA have been proposed to enhance robustness and accuracy. For instance, Chen et al. introduced filter bank CCA [21], multi-band filters to extract harmonic information, effectively enhancing the recognition performance, while Nakanishi et al. introduced task-related component analysis [22] to obtain the spatial filter by maximizing the inter-trial signal covariance, thereby further improving the classification accuracy. Although the CCA method and its extension have excellent performance, it overly relies on artificially designed reference signals, making it difficult to fully reflect the real EEG characteristics. Moreover, it is difficult to balance real-time performance and model complexity.

These above mentioned traditional SSVEP classification methods are theoretically mature and have clear implementation approaches. However, they fails to meet the processing requirements of complex EEG signals in terms of adaptability, generalization, and modeling capabilities. Therefore, there is an urgent need to introduce models with stronger representation and learning capabilities for improving and enhancing the SSVEP classification performance.

### 2.2. Deep Learning-Based SSVEP Classification

With the rapid development of deep learning, researchers have begun to attempt to apply it to EEG signal analysis, providing new ideas and technical means for SSVEP classification. A deep neural network can automatically extract discriminative features from large-scale, nonlinear, and high-dimensional raw signals. It can reduce the reliance on manual features, enhancing the accuracy, generalization ability, and real-time performance of classification models.

Lawhern et al. [23] proposed the EEGNet model combining deep convolution and separable convolution techniques, which demonstrates excellent generalization ability on multiple paradigm EEG datasets. Waytowich et al. [24] further verified the performance of EEGNet for the SSVEP classification. The results showed that it achieved an average cross-subject accuracy rate of nearly 80% in an SSVEP dataset containing 12 targets. Aravind Ravi et al. and Nakanishi et al. [25,26] proposed the C-CNN network structure combined with complex spectral information, which achieved a cross-subject accuracy rate of 81.60% and an in-subject accuracy rate of 92.33% on public datasets. Khok et al. and Wang et al. [27,28] designed a multi-task learning model based on dilated convolution, which can detect multiple frequencies at once. It achieved an accuracy rate of 92.20% on the Tsinghua benchmark dataset in a 1 s window width.

Recurrent neural networks (RNN) and their variants, long short-term memory networks (LSTMs), are suitable for modeling dynamic dependency structures in time series data, and can also be used for SSVEP classification. Pan et al. [29] proposed the SSVEPNET network, which integrated CNN and LSTM, and combined label smoothing and spectral normalization strategies, achieving an average accuracy rate of 84.45% in a 1 s window width. Zhang et al. [30] further designed a bidirectional LSTM network structure and introduced a correlation modeling mechanism. In a 0.8 s window and in a 1 s window, its accuracy rates reached 91.38% and 94.07%, respectively.

Recently, the introduction of transformer and attention mechanisms has brought new breakthroughs to SSVEP classification. Chen et al. [31] first applied the transformer architecture to SSVEP and proposed the SSVEPformer model, achieving better classification results than traditional CNN models in cross-subject experiments. Wan et al. [32] proposed GDNet-EEG introducing group deep convolution and channel attention mechanisms. It achieved accuracies of 84.11% and 85.93%, respectively, on the benchmark and BETA datasets, and reached 93.35% on the combined dataset of the two. Wang et al. [33] embedded the attention mechanism into the neural network structure, reducing the number of parameters while improving the information transmission efficiency. This model achieved an accuracy rate of 85.49% and an information transmission rate of 182.05 bits/min on the benchmark dataset in a 0.4 s window width.

In summary, deep learning has unequivocally proven its superiority over traditional methods in automatic feature learning and in handling complex signal variations. The diverse architectural innovations provide a rich foundation and strong motivation for our proposed hybrid model, which seeks to leverage the complementary strengths of unsupervised multi-channel modeling (i.e., MCRBM) and deep discriminative learning (i.e., CNN) to advance the state-of-the-art in SSVEP classification.

## 3. Methodology

### 3.1. Overall Framework

**Figure 1:**
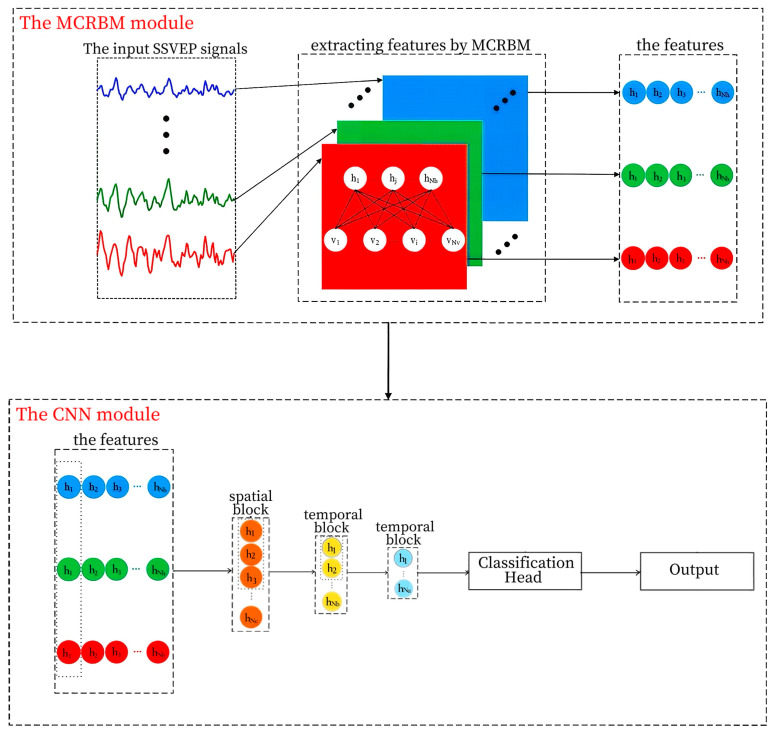
The structure of the MCRBM–CNN model.

The proposed MCRBM–CNN framework is designed as a hybrid deep learning architecture for robust SSVEP classification, comprising two principal modules: a multi-channel restricted Boltzmann machine (MCRBM) for unsupervised feature learning and a convolutional neural network (CNN) for discriminative spatiotemporal modeling and classification. The overall architecture is illustrated in Figure 1.

The input to the model is a two-dimensional raw EEG segment with dimensions [*C*, *T*], where *C* denotes the number of channels and *T* represents the number of temporal sampling points. This input is first processed by the MCRBM module to extract a compact, latent representation for each channel. The resulting feature maps are subsequently fed into the CNN module, which further abstracts both spatial and temporal patterns to produce the final classification output.

### 3.2. MCRBM Feature Extraction Module

**Figure 2:**
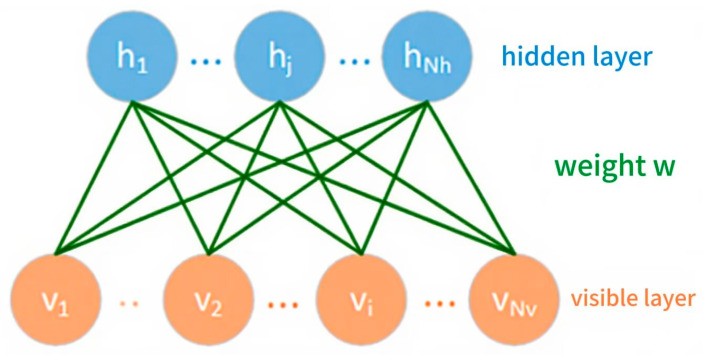
A model of the restricted Boltzmann machine.

The RBM is a typical generative neural network model belonging to the category of energy models. It is based on a probabilistic graphical model, and can model input data. Therefore, it is often used for feature extraction, data dimensionality reduction, and the pre-training of deep networks. As shown in Figure 2, the RBM is composed of two layers of neurons, namely a visible layer and a hidden layer, respectively. The lower layer is the visible layer containing *N_v_* nodes, which is used to receive the input data. The upper layer is the hidden layer containing *N_h_* nodes, which is used to capture the potential features of the data. The weight matrix $W\in R^{N_{v}\times N_{h}}$ connects the two layers.

Since the SSVEP signals are multi-channel and there are significant spatial correlations among different electrode channels for the entire EEG signal, therefore using a single-channel RBM to process the signal may cause parameter redundancy. To address this issue, we draw on the “weight sharing” concept from convolutional neural networks, and introduce the multi-channel restricted Boltzmann machine structure on the basis of the multi-RBM parallel structure. The MCRBM not only retains the feature extraction capability of the model but significantly reduces the number of parameters, thereby enhancing the training efficiency and generalization ability of the model.

Given the SSVEP signals containing *N_c_* channels, the input length of each channel is *N_v_*, and the hidden layer of the MCRBM is set to have *N_h_* neurons, then the input tensor of the model is $N_{c}\times N_{h}$ dimension, and the output tensor is dimension. In order to reduce the complexity, the MCRBM shared the weight matrix $W\in R^{N_{v}\times N_{h}}$ and bias vector $a\in R^{N_{v}}$ (for visible layer), $b\in R^{N_{h}}$ (for hidden layer) of each RBM among all channels, which can avoid the parameter redundancy and overfitting caused by the independent training of each channel. The energy function of the MCRBM model is defined as follows:

(1) $$E(v,h)=\sum_{k=1}^{N_{c}}\sum_{i=1}^{N_{v}}\frac{1}{2}v_{ki}^{2}-\sum_{k=1}^{N_{c}}\sum_{i=1}^{N_{v}}\sum_{j=1}^{N_{h}}v_{ki}w_{ij}h_{kj}-\sum_{k=1}^{N_{c}}\sum_{j=1}^{N_{h}}b_{j}h_{kj}-\sum_{k=1}^{N_{c}}\sum_{i=1}^{N_{v}}a_{i}v_{ki}$$

where $v_{ki}$ denotes the *i*th visible node of the *k*th channel, and $h_{kj}$ denotes the *j*th hidden node of the *k*th channel.

According to the energy function and the joint probability distribution of the RBM, the conditional probability distribution of multi-channel SSVEP signals can be obtained as follows:

(2) $$P(h_{kj}=1|v)=\sigma (\sum_{i=1}^{N_{v}}w_{ij}v_{ki}+b_{j})$$

(3) $$P(v_{ki}=1|h)=N(\sum_{j=1}^{N_{h}}w_{ij}h_{kj}+a_{i},1)$$

where σ(⋅) is a sigmoid function, N(⋅) is a normal distribution with a mean $\sum_{j=1}^{N_{h}}w_{ij}h_{kj}+a_{i}$ and a variance of 1. In other words, the hidden layer generated by the MCRBM conforms to the normal distribution.

The contrast divergence algorithm is used to train the model, and the values of the SSVEP signals are used as the initial state of the visible layer of the MCRBM. Firstly, the binary expressions of hidden layer neurons are obtained based on Equation (2), and then obtaining the probability that the value of visible layer neurons is 1 based on Equation (3) with these binary hidden layer neurons. As a result, we can obtain a set of hidden layer neurons and visible layer neurons. According to the gradient descent algorithm, the parameters of the MCRBM are updated as follows:

(4) $$\Delta w_{ij}=\alpha \left(\frac{1}{N_{c}}\sum_{k=1}^{N_{c}}\left(E_{data}\left[v_{ki}h_{kj}\right]-E_{\mathrm{mod}el}\left[v_{ki}h_{kj}\right]\right)\right)$$

(5) $$\Delta b_{j}=\alpha \left(\frac{1}{N_{c}}\sum_{k=1}^{N_{c}}\left(E_{data}[h_{kj}]-E_{\mathrm{mod}el}\left[h_{kj}\right]\right)\right)$$

(6) $$\Delta a_{i}=\alpha \left(\frac{1}{N_{c}}\sum_{k=1}^{N_{c}}\left(E_{data}\left[v_{ki}\right]-E_{\mathrm{mod}el}\left[v_{ki}\right]\right)\right)$$

where α is learning rate; $E_{data}[\cdot ]$ denotes the expected value of the energy function under the conditional probability distribution function p(v|h), representing the expected value of the energy function of the input SSVEP singles; similarly, $E_{\mathrm{mod}el}[\cdot ]$ denotes the expected value of the energy function under the conditional probability distribution function p(h|v), i.e., the expected value of the energy function of the SSVEP signal reconstructed by the MCRBM.

### 3.3. The CNN Classification Module

The feature representations generated by the MCRBM module are subsequently processed by a custom-designed CNN architecture to perform high level spatiotemporal feature extraction and classification. As shown in Figure 1, the CNN module is structured into three sequential components: a spatial feature extraction block, two temporal feature extraction blocks, and a classification head.

The spatial feature extraction block employs convolutional kernels of size C × 1 to perform feature learning across the channel dimension at individual time points. This operation effectively captures the spatial synchronicity and functional connectivity between different electrodes. The convolution is followed by batch normalization and an exponential linear unit (ELU) activation function to stabilize training and to alleviate the vanishing gradient problem.

The spatially enhanced feature maps are then passed through two temporal feature extraction blocks. Each block comprises a one-dimensional convolution (with kernel sizes 1 × *k*_1_ and 1 × *k*_2_, where *k*_1_ and *k*_2_ are the receptive field in the temporal direction) applied along the temporal dimension, followed by batch normalization, ELU activation, and max pooling. These layers are designed to identify local temporal patterns, periodicities, and morphological features characteristic of SSVEP responses at different stimulation frequencies.

The resulting feature tensor, enriched with both spatial and temporal information, is flattened and fed into the classification head. This consists of three fully connected (FC) layers. The first two FC layers employ ReLU activation functions for nonlinear transformation, while the final output layer uses a Softmax function to generate probability distributions over the target classes. The entire network is trained end-to-end using the cross-entropy loss function.

## 4. Experiment Settings

### 4.1. Datasets

To comprehensively evaluate the performance and generalization capability of the proposed MCRBM–CNN model, experiments were conducted on two publicly available SSVEP benchmark datasets exhibiting different characteristics in terms of target numbers, recording parameters, and experimental paradigms.

**Figure 3:**
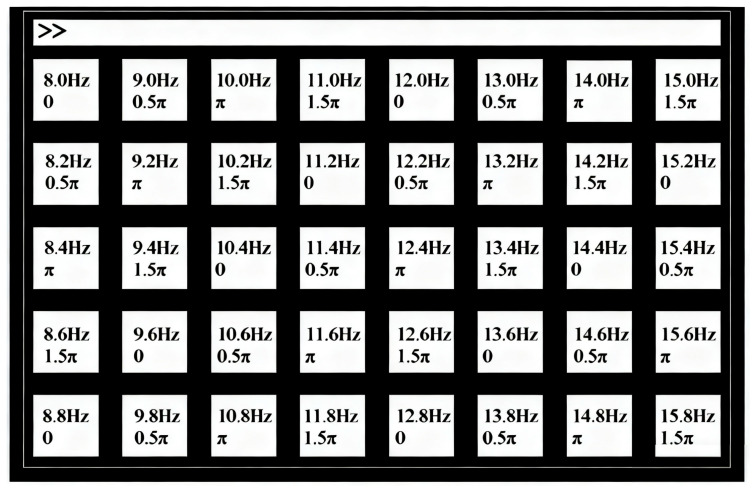
The visual stimulus editing interface of dataset I.

(1) Dataset I: The benchmark dataset introduced by Wang et al. [28] comprises SSVEP recordings from 35 healthy subjects focusing on 40 visual stimuli. The subjects were asked to shift their gaze to the target as soon as possible, and were instructed to avoid eye blinks during the stimulation duration. These targets adopt a frequency-phase hybrid coding method, specifically including 40 frequencies (8.0 Hz to 15.8 Hz, with a step size of 0.2 Hz) and 4 phases (0, 0.5π, π, 1.5π). As shown in Figure 3, all the targets are arranged in a 5×8 matrix layout.

Each subject participated in 6 experimental blocks, and each block contained 40 trials. In the stimulus timeline, each trial lasted for 6 s, including 0.5 s for the prompt, 5 s for the visual stimulation, and 0.5 s for rest. EEG signals were recorded using a Neuroscan Synamps 2 system with 64 electrodes configured in an extended 10–20 layout and a sampling frequency of 1000 Hz. The final sampling rate is 250 Hz (downsampled from the original 1000 Hz), and a 50 Hz notch filter was used to remove power frequency noises. Ultimately, the EEG signals of each subject were saved in the form of a four-dimensional tensor with dimensions of [64, 1500, 40, 6], representing the number of channels, the number of sampling points, the number of experiments, and the number of experimental blocks, respectively.

**Figure 4:**
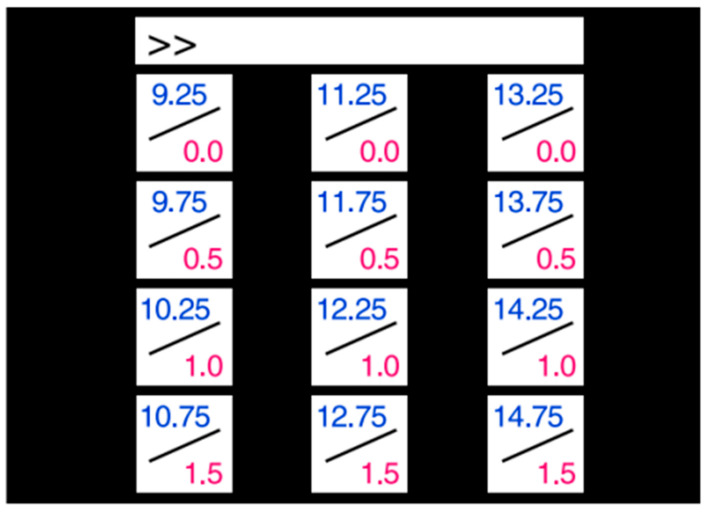
The visual stimulus editing interface of dataset II.

(2) Dataset II: The dataset provided by Nakanishi et al. [26] contains 12-class SSVEP data from 10 subjects, recorded in a simulated online BCI experiment. The 12 targets were arranged in a matrix and were distinguished using a combination of frequency and phase encoding. The visual stimulus editing interface is shown in Figure 4.

During the data collection process, each subject participated in 15 experimental blocks, and each block contained 12 trials. In each trial, the subjects were required to focus on a certain stimulus target for 4 s and then rest for 1 s to avoid fatigue. The subjects were asked to shift their gaze to the target within the same 1 s duration, and were asked to avoid eye blinks during the stimulation period to reduce eye movement artifacts. The data acquisition device is the BioSemi Active Two EEG system with an initial sampling rate of 2048 Hz. The start time of the visual stimulus is the 39th sampling point, which means there is 0.15 s of redundant time before the stimulus begins. The stimulus timeline includes three stages: 0–0.15 s, the redundant pre-stimulus period (excluding 39 sampling points); 0.15–4.15 s, the visual stimulation period; 4.15–5.0 s, the rest period. The collected EEG signals were processed through a 6–80 Hz band-pass filter, and the final sampling rate is 256 Hz (downsampled from the original 2048 Hz). The EEG signals of each subject were represented in a four-dimensional array with dimensions of [12, 8, 1114, 15], corresponding, respectively, to the number of targets, the number of channels, the number of sampling points, and the number of trial blocks.

The two datasets, respectively, represent the SSVEP experimental environments under different target numbers, sampling rates, and electrode configurations, and are suitable for verifying the generalization ability and classification performance of models in diverse data scenarios.

### 4.2. Preprocessing

For dataset I, first, 9 channels located in the parietal and occipital lobe regions were selected from the original 64 electrode channels: Pz, PO5, PO3, POz, PO4, PO6, O1, Oz, and O2. These channels have relatively high signal-to-noise ratios, and have been confirmed in previous studies to respond significantly to SSVEP signals, making them suitable as the input features for subsequent analysis. Next, the 6 experimental blocks and the 40 trials within each block were expanded and integrated into a 240 dimension complete trail, then each complete trail as an independent sample will be input into the model. Since the total duration of each trial is 6 s, the effective stimulation period is the middle 5 s. The initial 0.5 s and the final 0.5 s are prompts and rest periods, so they are excluded. The SSVEP signal has a 0.14 s latency due to optic nerve and cortical processing. Thus, the effective stimulation window starts at 0.5 s + 0.14 s = 0.64 s (relative to trial onset, or 0.14 s relative to stimulus onset at 0.5 s).

After the above processing, the EEG signals of each subject were transformed from a four-dimensional matrix with the original shape of [64, 1500, 40, 6] into a 240 two-dimensional matrix with the shape of [9, *T*], where *T* is the number of sampling points corresponding to the specified window time. Finally, to retain the typical SSVEP activation frequency band and to filter out low-frequency drift and high-frequency noise, a fourth-order band-pass filter was used to limit the data frequency range to 8–90 Hz.

For dataset II, its original data used a total of 8 channels, all of them located in the occipital and parietal regions, which met the SSVEP feature significance requirements. Therefore, all channels were retained for subsequent modeling. This dataset contains 15 blocks, and each block contains 12 trials, totaling 180 trials. Preprocessing requires that all trials be combined into independent samples. To obtain effective visual induction fragments, we performed cropping and segmentation according to the set window length to obtain two-dimensional fragmented samples with a shape of [8, *T*]. All samples are continuously segmented without overlapping or sliding windows; therefore, there is no potential temporal leakage or overlap between them. The value of *T* depends on the set time window, since the sampling rate of the original signal is 256 Hz. Similar to dataset I, a fourth-order band-pass filter was ultimately used to enhance the signal-to-noise ratio, limiting the signal frequency range to 8–90 Hz to eliminate eye movement, power line interference, and high-frequency noise, while retaining the main frequency components related to SSVEP. Consistent with dataset I, we excluded the first 0.14 s of the stimulation period to account for SSVEP latency. Thus, the effective stimulation window starts at 0.15 s + 0.14 s = 0.29 s (relative to trial onset, or 0.14 s relative to stimulus onset at 0.15 s).

### 4.3. Implementation and Parameter Configuration

The proposed MCRBM–CNN model was implemented using the PyTorch V2.9.1 deep learning framework. The MCRBM module was trained in an unsupervised manner using the contrastive divergence (CD-1) algorithm, while the entire hybrid architecture was subsequently fine-tuned end-to-end via backpropagation, minimizing the cross-entropy loss using the Adam optimizer with an initial learning rate of 0.001. The detailed parameter configuration for each layer is summarized in Table 1.

## 5. Results and Analysis

In order to evaluate the adaptability of the proposed model under different real-time requirements, we test it with different windows length (4 s, 2 s, and 1 s) on each dataset. Two kinds of typical methods are selected for performance comparison. One is the traditional algorithm FBCCA [21], which is based on band filtering and canonical correlation analysis. The other is EEGNet [24], a lightweight deep learning model. To ensure a rigorous and fair comparison, the hyperparameters for all models, including the baseline methods (FBCCA, EEGNet) and our proposed model, were optimized using a consistent strategy, specifically a grid search conducted on a held-out validation set. Key parameters, such as the number of FBCCA harmonics, learning rates, and regularization weights, were tuned to maximize the performance on this validation data. Furthermore, all models were evaluated under identical experimental conditions. They were trained and tested on the same dataset, utilizing the same set of EEG channels, identical preprocessing steps (including filtering and artifact handling), and the exact same data time windows.

Compared with these two methods, the classification ability and advantages of the proposed model under different conditions can be comprehensively demonstrated.

### 5.1. Performance Evaluation on Dataset I

#### 5.1.1. Model Performance Under a Window Length of 4 s on Dataset I

**Figure 5:**
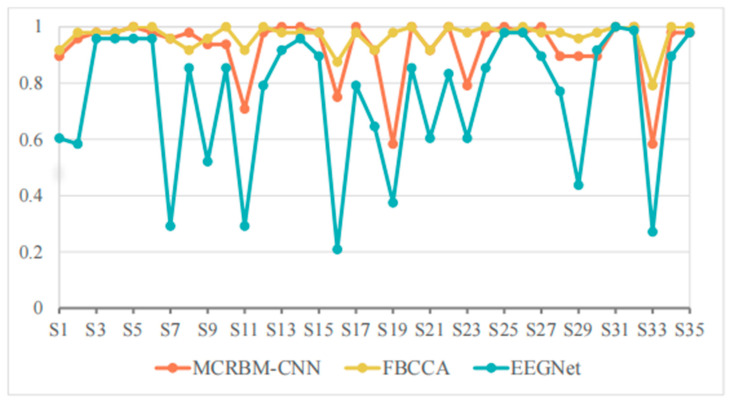
The accuracy change curves on dataset I under 4 s time windows.

The SSVEP classification accuracy of different models for 35 subjects from dataset I under a window length of 4 s is shown in Table 2 and Figure 5.

From Table 2 we can see that the MCRBM–CNN achieved an average classification accuracy of 92.61% when the window length is 4 s, which is better than the deep learning model EEGNet (75.08%) and close to the traditional optimized algorithm FBCCA (96.72%). This shows that the structure consisting of MCRBM and CNN has a strong ability to extract the spatial and temporal domain features of SSVEP signals, and can maintain good classification performance in high-dimensional multi-target tasks.

By analyzing the performance on each subject in detail, it can be found that for most subjects (such as S3, S4, S5, S14, S25, S31, S32, etc.), the three models can achieved high accuracy (all above 95%), and the performances are relatively stable. Typically, MCRBM–CNN achieves 100% accuracy for 10 subjects, showing its strong adaptability to parts of the data. Overall, based on the best performance of the individuals marked in bold in Table 2, the currently developed MCRBM–CNN model has achieved the best accuracy rate for 18 subjects, accounting for more than 50% of all involved participants. Although it cannot be compared with the traditional FBCCA that has been widely adopted in SSVEP-based scenarios, it marks a huge progress in feature extraction through deep learning. Especially, considering that for a few subjects (such as S11, S16, S19, and S33), the performances of the traditional EEGNet decreased significantly. For example, for S33, EEGNet’s accuracy was even lower at 27.08%, while MCRBM–CNN can achieve 58.33%. Taking into account that FBCCA is relatively robust and can maintain an accuracy of 79.17% on these samples, we infer that this may be due to the decreased performance of deep learning mechanisms. The potential subjects’ characteristics, such as the lower SNR and more artifacts, make it difficult for the neural network models to recognize typical SSVEP patterns during feature extraction.

Generally, the MCRBM–CNN model showed good stability and strong classification ability in relative wide-window scenes, while maintaining the advantages of neural network models for complex nonlinear features extraction, and achieved a certain balance between high accuracy and wide adaptability.

#### 5.1.2. Model Performance Under Window Length 2 s on Dataset I

**Figure 6:**
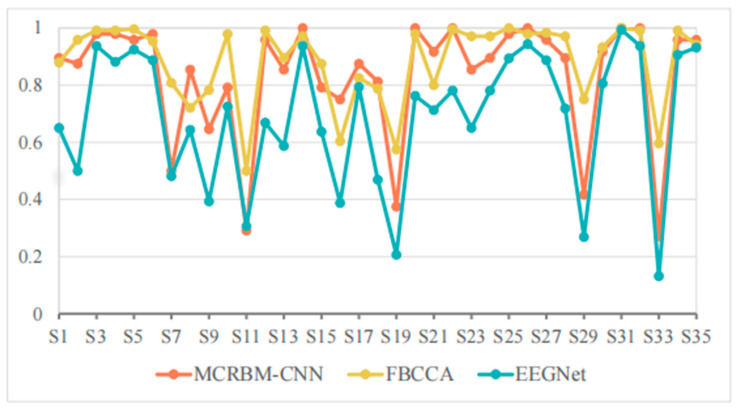
The accuracy change curves on dataset I under 2 s time windows.

Table 3 shows the classification accuracies of different models under a window length of 2 s on dataset I, and Figure 6 shows the visualization curve of the corresponding results.

As shown in Table 3, the average accuracy of FBCCA, EEGNet, and MCRBM–CNN are 88.40%, 68.93%, and 83.39%, respectively. We can see that, with the shortening of the time window, compared with the results under a window length of 4 s, the accuracy of all models decreased, while FBCCA still showed high stability and maintained a high accuracy level. The MCRBM–CNN model was rather superior to EEGNet, showing stronger feature extraction and modeling ability.

In terms of specific subjects, MCRBM–CNN achieved or approached 100% accuracy in the S14, S20, S22, S26, S31, and S32 samples. However, for some low-quality signals (such as S7, S11, S19, S29, and S33), the accuracy of the deep learning-based models of MCRBM–CNN and EEGNet decreased significantly. In particular, the accuracy of EEGNet in S33 was only 13.13%, while MCRBM–CNN can be about two-times higher with 27.08%. By comparison, FBCCA still maintained at 59.58%, reflecting the robustness of traditional algorithms for harmonic feature utilization. In fact, the advantage of FBCCA is its strong dependence on periodicity and harmonic components, so that it can make full use of the frequency domain information of SSVEP under a long time window, and still has a high classification accuracy under a medium time window of 2 s. However, MCRBM–CNN does not rely on frequency information and completes the modeling through automatic feature extraction, so it can be accepted and still maintained good performance for most samples, and even outperformed FBCCA for subjects S6, S8, S16, S17, S18, S23, and S21.

Nevertheless, MCRBM–CNN has the problem of low accuracy for S11, S19, S29, and S33 with below 50%, which indicates that the model still has a certain bottleneck of generalization when facing strong noise interference or atypical SSVEP samples.

In summary, MCRBM–CNN has strong competitiveness in SSVEP classification tasks with a relative medium window length, which is robust compared with traditional algorithms and better than the EEGNet model. However, the generalization ability for extreme samples still needs to be improved.

#### 5.1.3. Model Performance Under a Window Length of 1 s on Dataset I

**Figure 7:**
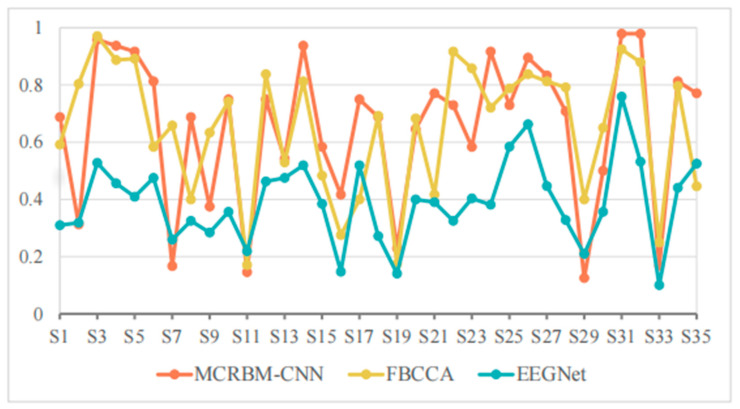
The accuracy change curves on dataset I under 1 s time windows.

Table 4 and Figure 7 show the accuracies of different models under a window length of 1 s on dataset I.

As shown in Table 4, the average accuracies of FBCCA, EEGNet, and MCRBM–CNN are 64.88%, 39.15%, and 65.12%, respectively. It can be seen that the performance of all models decreased significantly under a short window length. This is mainly because the too short time windows lack periodic features and harmonic information and reduce the signal-to-noise ratio, which affects the classification effect.

However, MCRBM–CNN was slightly higher than FBCCA on the whole, showing that it has certain modeling advantages for short-time SSVEP signals, especially without relying on frequency information, and it can still use multi-channel cooperative information for effective feature extraction. For example, its accuracies for subjects S3, S4, S5, S14, S24, S31, and S32 are higher than 90%, showing the strong adaptability and expressiveness of the MCRBM–CNN model.

By contrast, the EEGNet model was greatly interfered by short-term signals and performed poorly for most subjects. For example, the accuracies for S2, S7, S11, S19, S29, and S33 were less than 30%, and even dropped to 10.00% (e.g., subject S33), indicating that its generalization ability is limited in small sample and short window scenarios.

It is worth noting that FBCCA can still maintain more than 80% accuracy on multiple subjects, such as S2, S3, S12, S22, S23, S26, S27, S31, and S32, indicating stable performance. However, due to its strong dependence on harmonic components, the performance fluctuated greatly facing low quality or interference signals, such as the accuracies for S6, S17, S16, S17, S21, and S35, which were far lower than that of the developed MCRBM–CNN model. Statistically, the currently developed model has achieved the best accuracy rate among 20 subjects, accounting for more than 55%, which demonstrates the adaptability of the multi-channel weight sharing structure to low signal-to-noise ratio SSVEP signals.

### 5.2. Performance Evaluation on Dataset II

#### 5.2.1. Model Performance Under a Window Length of 4 s on Dataset II

**Figure 8:**
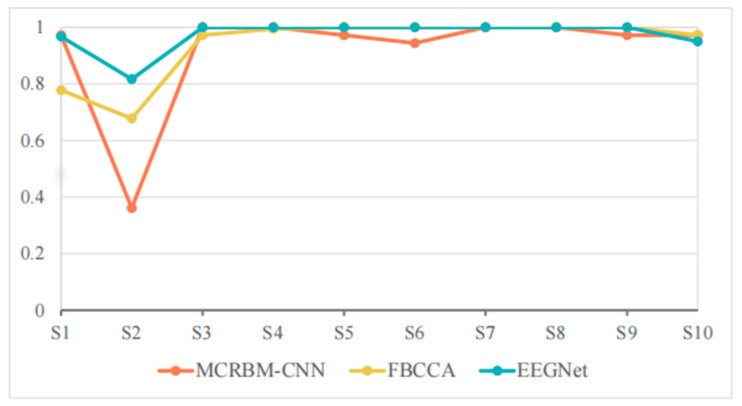
The accuracy change curves on dataset II under 4 s time windows.

The classification accuracies of different models under a window length of 4 s for 10 subjects from dataset II are shown in Table 5, and Figure 8.

As we see, EEGNet achieved extremely high accuracy for most subjects, and even achieved 100% accuracy for subjects S3–S8. FBCCA also performed well for S5–S9 with 100% accuracy and approached or even exceeded 97% accuracy for S3, S4, and S10. By comparison, the classification accuracy of MCRBM–CNN remained at a high level for most subjects for S1, S3, S4, S7, S8, and S10. However, it decreased significantly for individual subject S2 with 36.11%.

In general, when a window length is 4 s, all models can effectively extract the features of SSVEP signals and complete the classification. EEGNet achieved an average accuracy of 97.33% in 4 s windows, surpassing MCRBM–CNN (91.94%) and FBCCA (93.94%). This advantage stems from EEGNet’s compact, supervised convolutional design, which excels in simpler tasks (fewer targets, cleaner signals) with sufficient data for supervised feature learning.

#### 5.2.2. Model Performance Under a Window Length of 2 s on Dataset II

**Figure 9:**
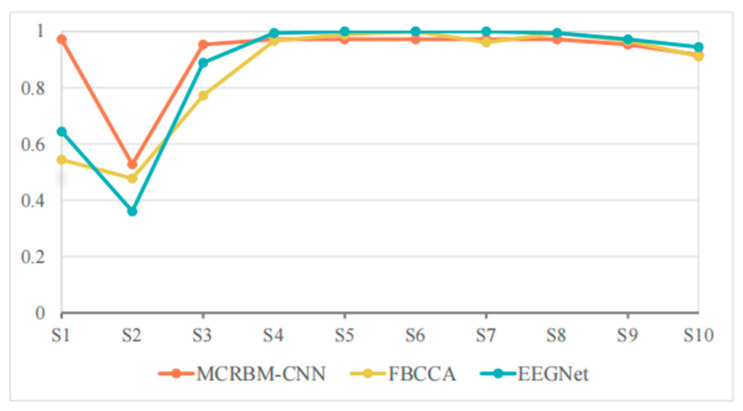
The accuracy change curves on dataset II under 2 s time windows.

Table 6 and Figure 9 shows the classification accuracies of different models under a window length of 2 s on dataset II. For the overall performance, although slightly lower than its performance when the window length is 4 s, MCRBM–CNN maintained a stable performance with the average accuracy of 91.85% under a window length of 2 s, which is better than EEGNet (88.00%) and FBCCA (85.93%).

For the specific performance of each subject, S1 and S2 are the subjects with high discrimination of the three models. The classification accuracy of MCRBM–CNN for S1 still maintained at a high level of 97.22%, while the accuracies of FBCCA (54.44%) and EEGNet (64.44%) decreased significantly, indicating that they are sensitive to signal length variations. Subject S2 is the trough of all accuracies, especially the performance of EEGNet for this subject is obviously limited. Considering that, even with the longer time windows of 4 s, the accuracy is also relatively low, we infer that this may be affected by the individual state of the subject or the signal quality.

For the subjects S3 to S10, the accuracies of the three models maintained at a high level, especially EEGNet, which was almost saturated in some subjects. MCRBM–CNN also performed stably, basically maintaining a similar performance to a window length of 4 s. Although FBCCA performed well for most subjects, the accuracy for S3 decreased significantly.

In summary, MCRBM–CNN showed strong stability, adaptability, and generalization ability under a window length of 2 s, which is one of the more ideal models in this condition. EEGNet performed well but fluctuated slightly, while FBCCA was more suitable for application in scenarios with a longer window length or a higher signal quality.

#### 5.2.3. Model Performance Under a Window Length of 1 s on Dataset II

**Figure 10:**
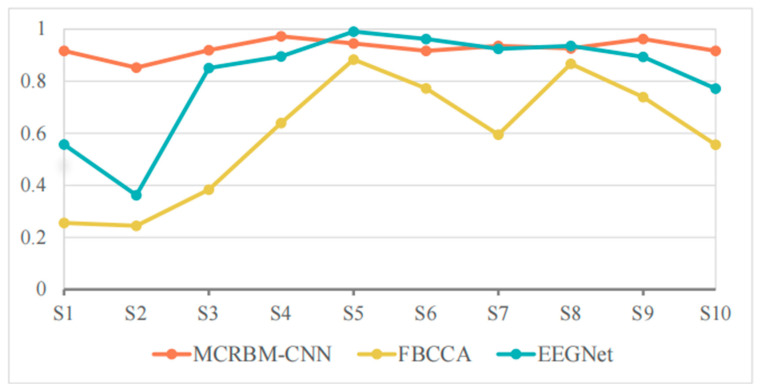
The accuracy change curves on dataset II under 1 s time windows.

Table 7 and Figure 10 show the classification accuracies of different models under a window length of 1 s on dataset II. For the perspective of overall performance, MCRBM–CNN still demonstrated excellent stability under this short window length, maintaining a high average accuracy rate (92.61%), and ranking first among all models. EEGNet followed closely, with its accuracy of 81.40% decreased significantly compared to the long window length. The performance of FBCCA was significantly affected under the short window length, with a significant drop in average accuracy to 59.33%.

For the specific subjects, S1 and S2 represent the samples with the most significant differences in the performance of each model. The MCRBM–CNN model still maintained a good classification effect for the two subjects, while the accuracies of FBCCA and EEGNet significantly decreased, indicating that their processing capabilities for short-term SSVEP signals are limited. Especially for FBCCA, its accuracy was at a relatively low level for multiple subjects, demonstrating its sensitivity to the shortening of signal length.

Although the EEGNet model achieved relatively high accuracy for some subjects (such as S5, S6, and S8), the overall accuracy showed slight fluctuations. By contrast, the accuracy of MCRBM–CNN was more stable, with only a few subjects (such as S2) performing slightly worse, while remaining at a high level for most subjects.

In conclusion, the most prominent strength of MCRBM–CNN is its ability to maintain high accuracy when window lengths are shortened (1 s–2 s), a critical requirement for real-time BCI applications. Particularly in subjects with relatively poor signal quality, its robustness advantage was more pronounced.

### 5.3. The Ablation Experiments

To further verify the performance of MCRBM–CNN, we conducted the ablation experiments on both datasets. The MCRBM-only method extracts features from the original SSVEP signals with the MCRBM module, then classifies them with traditional neural network. The CNN-only method directly classifies the SSVEP signals using the CNN module without feature extraction. The MCRBM-only and CNN-only methods use the same parameters and settings as the MCRBM–CNN method. The ablation experiment results under different window lengths on two datasets are shown in Table 8 and Table 9, respectively.

As shown in Table 8 and Table 9, the CNN-only model performed the worst in most scenarios, indicating that when there is a lack of preprocessing of unsupervised features in the early stage, directly conducting discriminative modeling is difficult to effectively extract the key features of SSVEP. Although the MCRBM-only model outperforms the CNN-only model, it lacks subsequent deepening of spatiotemporal features, resulting in limited classification accuracy.

The classification performance of the MCRBM–CNN hybrid model is significantly superior to that of the individual MCRBM module or CNN module. Especially in the short time window (1 s) scenario, the advantage is more prominent. This verifies the synergistic effect of the unsupervised feature extraction of MCRBM and the discriminative modeling of CNN.

### 5.4. The Computational Efficiency

To evaluate the computational efficiency of different models, we recorded the time taken by each model to classify a sample in Table 10. All the experiments are conducted on AMD Ryzen 7-5800H CPU, NVIDIA GeForce RTX 3060 Laptop GPU, Windows 10 OS, Python 3.8.20, Cuda 11.8, and NumPy 1.24.0.

As shown in Table 10, the FBCCA clearly requires a much longer time to classify a sample. This is because that FBCCA needs to conduct a typical canonical correlation analysis with all SSVEP signals when classifying a test sample. By contrast, both EEGNet and MCRBM–CNN are based on machine learning, they can directly classify test samples with the already-trained model, there is no need to train the model every time. Therefore, they are significantly faster than the FBCCA method. Since MCRBM–CNN has an additional unsupervised feature extraction process compared to EEGNet, its running time is slightly longer than that of EEGNet. Although MCRBM–CNN is not the fastest method, the time needed to classify a sample is only 0.054~0.086 s, which is enough for real-time application in most situations.

## 6. Discussions

### 6.1. The Neurophysiological Interpretability

To provide neurophysiological insight into the decisions of our MCRBM–CNN model, we conducted a post hoc analysis to identify the features that most contributed to its classification performance. We found that our model autonomously learned to prioritize contributions from occipital and parieto-occipital channels (e.g., O1, Oz, O2, POz), which aligns precisely with the known neuroanatomy of the visual cortex responsible for generating SSVEPs.

Furthermore, an analysis of the model’s spectral focus revealed a strong weighting not only on the fundamental stimulation frequencies but on their second harmonics. This demonstrates that the model effectively leverages the nonlinear harmonic components of the SSVEP response, a well-established physiological phenomenon, to improve discriminability. This inherent capability to exploit harmonic information is a key factor in its robust performance, particularly under the challenging conditions of short time windows where the signal-to-noise ratio is lower. This analysis confirms that our model’s high performance is not merely a statistical artifact but is grounded in a physiologically plausible processing mechanism.

### 6.2. The Limitations of the Current Research

While the proposed MCRBM–CNN model demonstrated strong classification performance, it is important to note that these results were derived from a subject-dependent evaluation. This approach may overestimate its efficacy when applied to new, unseen subjects in real-world scenarios, as the model was trained and tested on data from the same individuals. To enhance the model’s generalizability and practical application potential, future work will focus on expanding the participant cohort and incorporating subject-independent validation schemes, such as leave-one-subject-out cross-validation. Additionally, we will explore domain adaptation methods to improve cross-subject robustness and to facilitate deployment in clinical BCI systems.

Meanwhile, while this study established comparisons against standard benchmarks, future work will include a more extensive evaluation against a wider array of state-of-the-art deep learning architectures, such as a compact CNN, to further validate the advantages of the proposed framework.

It is also important to acknowledge our limitations regarding statistical validation and practical BCI metrics. The current analysis primarily relied on mean accuracy and variance to compare performance across conditions. A more rigorous statistical evaluation, employing paired statistical tests (e.g., paired t-tests or non-parametric equivalents) across subjects, is required to definitively establish the significance of the observed improvements, particularly for modest performance gains in longer time windows. Furthermore, to better quantify the practical utility and information efficiency of the system, a critical aspect for real-world BCI applications, metrics such as the information transfer rate (ITR) must be computed and analyzed across different stimulus durations. In our immediate future work, we will conduct this comprehensive analysis, integrating significance testing and ITR calculations to provide a more robust and application-oriented validation of the method’s advantages.

### 6.3. The Practicability in Real-Time BCI Applications

Generally, according to the BCI real-time standards, if the latency < 0.2 s, it can smoothly interact between human and computer. The related analysis confirms that the proposed MCRBM–CNN framework possesses the low computational latency required for real-time BCI applications. However, the current implementation relies on a standard GPU server. For widespread, practical deployment in mobile or clinical settings, future work must focus on model optimization for lightweight, low-power hardware. Our subsequent research will therefore investigate techniques for model compression and efficient deployment on wearable or embedded systems to bridge the gap between laboratory-grade performance and real-world usability.

## 7. Conclusions

Based on the multi-channel restricted Boltzmann machine and convolutional neural network, this study proposed a hybrid SSVEP classification framework. First, we designed a shared-weight MCRBM module, which enabled each channel to independently learn hidden features, thereby achieving feature dimension compression and noise suppression. Then, we constructed a CNN module which extracted the spatial relationships among different channels through a spatial convolution block, and extracted the dynamic change characteristics of the SSVEP signals in the time dimension with two cascaded temporal convolution blocks. Then, a fully connected layer was used to complete the classification of the SSVEP signals. The results of multiple experiments on two datasets showed that the proposed MCRBM–CNN model achieved excellent performance under the conditions of less window lengths of all used datasets, indicating the model has good stability for the SSVEP classification under extreme conditions.

In the future, we will further optimize the structure and training strategy of the MCRBM–CNN model, extend it to other types of EEG signal classification tasks, and explore the fusion capability of MCRBM–CNN in multimodal BCI systems.

## Figures and Tables

**Table 1:** The main network parameters of the MCRBM–CNN model.

Module	Type of Layer	Parameters Setting
MCRBM	visible layer	the length of each channel: *T*
	hidden layer	the number of hidden nodes in each channel: 256
CNN	spatial feature block	Conv2d, kernel size = C × 1, filters = C BatchNorm2d, ELU activation function
	temporal feature block #1	Conv2d, kernel size = 1 × 4, filters = 2C BatchNorm2d, ELU activation function Maxpool2d, kernel size = 1 × 2
	temporal feature block #2	Conv2d, kernel size = 1 × 2, filters = 2C BatchNorm2d, ELU activation function Maxpool2d, kernel size = 1 × 2
	classification head	three fully connected layer, the dimensions are 512, 128, *N*, respectively, ReLU activation function

**Table 2:** The SSVEP classification accuracy on dataset I under 4 s time windows.

Subjects Number	FBCCA	EEGNet	MCRBM–CNN
S1	0.9167	0.6042	0.8958
S2	0.9792	0.5833	0.9583
S3	0.9792	0.9583	0.9792
S4	0.9792	0.9583	0.9792
S5	1.0000	0.9583	1.0000
S6	1.0000	0.9583	0.9792
S7	0.9583	0.2917	0.9583
S8	0.9167	0.8542	0.9792
S9	0.9583	0.5208	0.9375
S10	1.0000	0.8542	0.9375
S11	0.9167	0.2917	0.7083
S12	1.0000	0.7917	0.9792
S13	0.9792	0.9167	1.0000
S14	0.9792	0.9583	1.0000
S15	0.9792	0.8958	0.9792
S16	0.8750	0.2083	0.7500
S17	0.9792	0.7917	1.0000
S18	0.9167	0.6458	0.9167
S19	0.9792	0.3750	0.5833
S20	1.0000	0.8542	1.0000
S21	0.9167	0.6042	0.9167
S22	1.0000	0.8333	1.0000
S23	0.9792	0.6042	0.7917
S24	1.0000	0.8542	0.9792
S25	0.9792	0.9792	1.0000
S26	1.0000	0.9792	0.9792
S27	0.9792	0.8958	1.0000
S28	0.9792	0.7708	0.8958
S29	0.9583	0.4375	0.8958
S30	0.9792	0.9167	0.8958
S31	1.0000	1.0000	1.0000
S32	1.0000	0.9875	1.0000
S33	0.7917	0.2708	0.5833
S34	1.0000	0.8958	0.9792
S35	1.0000	0.9792	0.9792
Average	0.9672	0.7508	0.9261
Variance	0.0019	0.0589	0.0123

**Table 3:** The SSVEP classification accuracy on dataset I under 2 s time windows.

Subjects Number	FBCCA	EEGNet	MCRBM–CNN
S1	0.8792	0.6500	0.8958
S2	0.9583	0.5000	0.8750
S3	0.9917	0.9375	0.9792
S4	0.9917	0.8812	0.9792
S5	0.9958	0.9250	0.9583
S6	0.9542	0.8875	0.9792
S7	0.8083	0.4813	0.5000
S8	0.7208	0.6438	0.8542
S9	0.7833	0.3937	0.6458
S10	0.9792	0.7250	0.7917
S11	0.5000	0.3063	0.2917
S12	0.9917	0.6687	0.9583
S13	0.8958	0.5875	0.8542
S14	0.9708	0.9375	1.0000
S15	0.8750	0.6375	0.7917
S16	0.6042	0.3875	0.7500
S17	0.8250	0.7937	0.8750
S18	0.7875	0.4688	0.8125
S19	0.5750	0.2062	0.3750
S20	0.9792	0.7625	1.0000
S21	0.8000	0.7125	0.9167
S22	0.9958	0.7812	1.0000
S23	0.9708	0.6500	0.8542
S24	0.9708	0.7812	0.8958
S25	1.0000	0.8938	0.9792
S26	0.9792	0.9437	1.0000
S27	0.9833	0.8875	0.9583
S28	0.9708	0.7188	0.8958
S29	0.7500	0.2687	0.4167
S30	0.9333	0.8063	0.9167
S31	1.0000	0.9938	1.0000
S32	0.9917	0.9375	1.0000
S33	0.5958	0.1313	0.2708
S34	0.9917	0.9062	0.9583
S35	0.9417	0.9313	0.9583
Average	0.8840	0.6893	0.8339
Variance	0.0199	0.0560	0.0444

**Table 4:** The SSVEP classification accuracy on dataset I under 1 s time windows.

Subject Number	FBCCA	EEGNet	MCRBM–CNN
S1	0.5917	0.3094	0.6875
S2	0.8042	0.3187	0.3125
S3	0.9708	0.5281	0.9583
S4	0.8875	0.4562	0.9375
S5	0.8917	0.4094	0.9167
S6	0.5833	0.4750	0.8125
S7	0.6583	0.2594	0.1667
S8	0.4000	0.3250	0.6875
S9	0.6333	0.2844	0.3750
S10	0.7417	0.3563	0.7500
S11	0.1708	0.2188	0.1458
S12	0.8375	0.4625	0.7500
S13	0.5292	0.4750	0.5417
S14	0.8125	0.5188	0.9375
S15	0.4833	0.3844	0.5833
S16	0.2750	0.1469	0.4167
S17	0.4000	0.5188	0.7500
S18	0.6917	0.2719	0.6875
S19	0.1750	0.1406	0.2292
S20	0.6833	0.4000	0.6458
S21	0.4167	0.3906	0.7708
S22	0.9167	0.3250	0.7292
S23	0.8583	0.4031	0.5833
S24	0.7208	0.3812	0.9167
S25	0.7875	0.5844	0.7292
S26	0.8375	0.6625	0.8958
S27	0.8125	0.4469	0.8333
S28	0.7917	0.3281	0.7083
S29	0.4000	0.2094	0.1250
S30	0.6500	0.3563	0.5000
S31	0.9250	0.7594	0.9792
S32	0.8792	0.5312	0.9792
S33	0.2500	0.1000	0.1667
S34	0.7958	0.4406	0.8125
S35	0.4458	0.5250	0.7708
Average	0.6488	0.3915	0.6512
Variance	0.0521	0.0207	0.0678

**Table 5:** The SSVEP classification accuracy on dataset II under 4 s time windows.

Subject Number	FBCCA	EEGNet	MCRBM–CNN
S1	0.7778	0.9667	0.9722
S2	0.6778	0.8167	0.3611
S3	0.9722	1.0000	1.0000
S4	0.9944	1.0000	1.0000
S5	1.0000	1.0000	0.9722
S6	1.0000	1.0000	0.9444
S7	1.0000	1.0000	1.0000
S8	1.0000	1.0000	1.0000
S9	1.0000	1.0000	0.9722
S10	0.9722	0.9500	0.9722
Average	0.9394	0.9733	0.9194
Variance	0.0131	0.0033	0.0388

**Table 6:** The SSVEP classification accuracy on dataset II under 2 s time windows.

Subject Number	FBCCA	EEGNet	MCRBM–CNN
S1	0.5444	0.6444	0.9722
S2	0.4778	0.3611	0.5278
S3	0.7722	0.8889	0.9537
S4	0.9667	0.9944	0.9722
S5	0.9889	1.0000	0.9722
S6	1.0000	1.0000	0.9722
S7	0.9611	1.0000	0.9722
S8	0.9944	0.9944	0.9722
S9	0.9667	0.9722	0.9537
S10	0.9111	0.9444	0.9167
Average	0.8583	0.8800	0.9185
Variance	0.0381	0.0452	0.0192

**Table 7:** The SSVEP classification accuracy on dataset II under 1 s time windows.

Subject Number	FBCCA	EEGNet	MCRBM–CNN
S1	0.2556	0.5571	0.9167
S2	0.2444	0.3619	0.8519
S3	0.3833	0.8500	0.9190
S4	0.6389	0.8952	0.9722
S5	0.8833	0.9905	0.9444
S6	0.7722	0.9619	0.9167
S7	0.5944	0.9238	0.9352
S8	0.8667	0.9357	0.9259
S9	0.7389	0.8929	0.9619
S10	0.5556	0.7714	0.9167
Average	0.5933	0.8140	0.9261
Variance	0.0550	0.0406	0.0011

**Table 8:** The ablation experiments on dataset I.

Subjects Number	MCRBM-Only			CNN-Only			MCRBM–CNN		
	4 s	2 s	1 s	4 s	2 s	1 s	4 s	2 s	1 s
S1	0.6875	0.7083	0.5208	0.6250	0.5625	0.4167	0.8958	0.8958	0.6875
S2	0.5208	0.6458	0.3333	0.5833	0.4792	0.2292	0.9583	0.8750	0.3125
S3	0.7292	1.0000	0.8750	0.8542	1.0000	0.6042	0.9792	0.9792	0.9583
S4	1.0000	0.9375	0.9375	0.8750	0.8125	0.6667	0.9792	0.9792	0.9375
S5	1.0000	0.8958	0.7917	0.8542	0.8333	0.6667	1.0000	0.9583	0.9167
S6	0.5625	0.6458	0.5208	0.8958	0.8125	0.6042	0.9792	0.9792	0.8125
S7	0.8333	0.5417	0.1875	0.5625	0.2750	0.2917	0.9583	0.5000	0.1667
S8	1.0000	0.7708	0.5208	0.7292	0.5833	0.4375	0.9792	0.8542	0.6875
S9	0.8125	0.3542	0.2708	0.4583	0.4167	0.2500	0.9375	0.6458	0.3750
S10	0.9167	0.7083	0.6042	0.7083	0.5833	0.4792	0.9375	0.7917	0.7500
S11	0.4375	0.2083	0.1458	0.5417	0.2500	0.1250	0.7083	0.2917	0.1458
S12	0.6667	0.7708	0.4792	0.7500	0.6667	0.4583	0.9792	0.9583	0.7500
S13	0.5833	0.7500	0.6042	0.9375	0.6458	0.3333	1.0000	0.8542	0.5417
S14	1.0000	1.0000	0.8333	1.0000	1.0000	0.9167	1.0000	1.0000	0.9375
S15	1.0000	0.8750	0.7708	0.6875	0.5625	0.3125	0.9792	0.7917	0.5833
S16	0.7292	0.4792	0.4167	0.6458	0.3542	0.2708	0.7500	0.7500	0.4167
S17	0.7083	0.6250	0.5625	0.6250	0.6458	0.5000	1.0000	0.8750	0.7500
S18	0.6458	0.5208	0.4792	0.7083	0.5208	0.4792	0.9167	0.8125	0.6875
S19	0.4792	0.3333	0.2500	0.2083	0.2500	0.2292	0.5833	0.3750	0.2292
S20	1.0000	0.9792	0.6042	0.7083	0.6250	0.4375	1.0000	1.0000	0.6458
S21	0.7292	0.7708	0.6875	0.7083	0.7708	0.4375	0.9167	0.9167	0.7708
S22	1.0000	0.9167	0.6875	0.8958	0.7292	0.4375	1.0000	1.0000	0.7292
S23	0.4792	0.5208	0.3958	0.6667	0.7292	0.2292	0.7917	0.8542	0.5833
S24	1.0000	0.9792	0.8958	0.8750	0.7500	0.6458	0.9792	0.8958	0.9167
S25	0.7083	0.6458	0.4792	0.8750	0.8333	0.5625	1.0000	0.9792	0.7292
S26	1.0000	1.0000	0.8542	0.9375	1.0000	0.6042	0.9792	1.0000	0.8958
S27	0.9583	0.8958	0.7292	0.8542	0.8542	0.5625	1.0000	0.9583	0.8333
S28	0.8125	0.8125	0.6458	0.6667	0.7083	0.4375	0.8958	0.8958	0.7083
S29	0.8333	0.3542	0.2292	0.5208	0.4375	0.2500	0.8958	0.4167	0.1250
S30	1.0000	0.8750	0.4375	0.8075	0.7708	0.3333	0.8958	0.9167	0.5000
S31	1.0000	1.0000	0.8750	1.0000	1.0000	0.7708	1.0000	1.0000	0.9792
S32	1.0000	1.0000	0.8542	0.9792	0.9583	0.6250	1.0000	1.0000	0.9792
S33	0.3125	0.2500	0.1250	0.3542	0.2708	0.1042	0.5833	0.2708	0.1667
S34	1.0000	0.8750	0.6458	0.8958	0.8542	0.4167	0.9792	0.9583	0.8125
S35	1.0000	1.0000	0.6250	0.9167	0.9792	0.5625	0.9792	0.9583	0.7708
Average	0.8042	0.7327	0.5679	0.7409	0.6721	0.4482	0.9261	0.8339	0.6512

**Table 9:** The ablation experiments on dataset II.

Subjects Number	MCRBM-Only			CNN-Only			MCRBM–CNN		
	4 s	2 s	1 s	4 s	2 s	1 s	4 s	2 s	1 s
S1	0.8519	0.7222	0.6852	0.6759	0.7870	0.7222	0.9722	0.9722	0.9167
S2	0.5833	0.5741	0.5278	0.4375	0.3333	0.4630	0.3611	0.5278	0.8519
S3	0.7778	0.7222	0.5000	0.8889	0.7778	0.6944	1.0000	0.9537	0.9190
S4	0.9722	0.9444	1.0000	0.9444	0.9722	0.8611	1.0000	0.9722	0.9722
S5	0.8889	0.8333	0.9722	0.8333	0.8611	0.6944	0.9722	0.9722	0.9444
S6	1.0000	0.9722	0.9444	1.0000	0.9444	0.9444	0.9444	0.9722	0.9167
S7	0.9444	0.9167	0.8611	0.8611	0.8889	0.9167	1.0000	0.9722	0.9352
S8	1.0000	1.0000	1.0000	1.0000	1.0000	0.9722	1.0000	0.9722	0.9259
S9	0.8333	0.6944	0.7778	0.9167	0.8611	0.6389	0.9722	0.9537	0.9619
S10	0.6944	0.7315	0.6944	0.5556	0.4907	0.4630	0.9722	0.9167	0.9167
Average	0.8546	0.8111	0.7963	0.8113	0.7917	0.7370	0.9194	0.9185	0.9261

**Table 10:** The time taken by each model to classify a sample (units: s).

Model	Dataset I			Dataset II		
	4 s	2 s	1 s	4 s	2 s	1 s
FBCCA	69.671	64.694	60.331	21.880	20.423	19.385
EEGNet	0.072	0.066	0.041	0.044	0.048	0.040
MCRBM–CNN	0.086	0.079	0.054	0.062	0.061	0.057

## Data Availability

The original contributions presented in this study are included in the article. Further inquiries can be directed to the corresponding author.
